# Supplementary material for: Combination of machine learning and data envelopment analysis to measure the efficiency of the Tax Service Office
Source: PeerJ Comput Sci. 2025 Feb 17;11:e2672. doi: 10.7717/peerj-cs.2672 (PMC11888853; doi:10.7717/peerj-cs.2672)
Supplement: Supplemental Information 8 [file peerj-cs-11-2672-s008.pdf]

Table A1. Ideal dataset.

DMU	Input	Output
A1	100	100
...	...	...
A25	100	100
A26	100	1,000,000
...	...	...
A50	100	1,000,000
A51	1,000,000	100
...	...	...
A75	1.000.000	100
A76	1,000,000	1,000,000
...	...	...
A100	1,000.000	1,000.000
