# Supplementary material for: Combination of machine learning and data envelopment analysis to measure the efficiency of the Tax Service Office
Source: PeerJ Comput Sci. 2025 Feb 17;11:e2672. doi: 10.7717/peerj-cs.2672 (PMC11888853; doi:10.7717/peerj-cs.2672)
Supplement: Supplemental Information 9 [file peerj-cs-11-2672-s009.pdf]

**Table A2.** Historical dataset.

| **DMU** | **Vin1** | **Vin2** | **...** | **Vin7** | **Vout1** | **Vout2** | **...** | **Vout6** |
|---|---|---|---|---|---|---|---|---|
| CQL | 36 | 4297 | ... | 7211834457 | 1.0306 | 138.82 | ... | 185 |
| EXA | 50 | 6610 | ... | 9785803301 | 1.0484 | ... | 2243 | 586 |
| ... | ... | ... | ... | ... | ... | ... | ... | ... |
| WOO | 43 | 6970 | ... | 9611797295 | 1.1838 | ... | 1694 | 362 |
