# Supplementary material for: Combination of machine learning and data envelopment analysis to measure the efficiency of the Tax Service Office
Source: PeerJ Comput Sci. 2025 Feb 17;11:e2672. doi: 10.7717/peerj-cs.2672 (PMC11888853; doi:10.7717/peerj-cs.2672)
Supplement: Supplemental Information 10 [file peerj-cs-11-2672-s010.pdf]

Table A3. Columns list and description.

Column	Unit	Description
DMU	-	Masked tax serivice office code
Vin1	taxpayer	Number of Treasury Taxpayers
Vin2	taxpayer	Number of corporate taxpayers
Vin3	taxpayer	Number of individual taxpayers (employees)
Vin4	taxpayer	Number of non-employee taxpayers
Vin5	employee	Number of Tax Auditors
Vin6	employee	Number of Account Representatives
Vin7	IDR	Budget realization amount
Vout1	percent	Compliance rate of annual tax return submission
Vout2	percent	Percentage of revenue achievement
Vout3	percent	Percentage of revenue growth achievement
Vout4	letter	Number of issued tax advisories
Vout5	letter	Number of tax advisories that have been paid
Vout6	tax audit	Number of completed tax audits
