# Supplementary material for: Combination of machine learning and data envelopment analysis to measure the efficiency of the Tax Service Office
Source: PeerJ Comput Sci. 2025 Feb 17;11:e2672. doi: 10.7717/peerj-cs.2672 (PMC11888853; doi:10.7717/peerj-cs.2672)
Supplement: Supplemental Information 11 [file peerj-cs-11-2672-s011.pdf]

Table A4. Statistical description.

Variable	count	mean	std	min	25%	50%	75%	max
Vin1	352	416	508	-	42	257	632	4.995
Vin2	352	6.056	3.503	295	3.270	5.637	8.226	22.320
Vin3	352	38.391	26.517	-	20.347	38.635	53.784	123.848
Vin4	352	13.224	10.308	-	5.316	12.542	19.436	56.942
Vin5	352	15	9	3	7	14	20	49
Vin6	352	32	7	10	26	32	36	54
Vin7	352	10.158	2.700	5.819	8.604	9.680	10.978	23.270
Vout1	352	104	7	76	101	102	106	147
Vout2	352	125	13	102	117	123	132	173
Vout3	352	40	37	(22)	19	31	51	293
Vout4	352	1.497	823	173	916	1.335	1.912	4.682
Vout5	352	1.351	734	94	806	1.253	1.776	5.107
Vout6	352	355	164	101	249	329	438	1.910
