# Supplementary material for: Combination of machine learning and data envelopment analysis to measure the efficiency of the Tax Service Office
Source: PeerJ Comput Sci. 2025 Feb 17;11:e2672. doi: 10.7717/peerj-cs.2672 (PMC11888853; doi:10.7717/peerj-cs.2672)
Supplement: Supplemental Information 12 [file peerj-cs-11-2672-s012.pdf]

Table A5. Skewness.

Vin1	Vin2	...	Vin7	Vout1	Vout2	...	Vout6
3.18	0.81	...	2.27	1.04	1.1	...	2.84
