# Supplementary material for: Combination of machine learning and data envelopment analysis to measure the efficiency of the Tax Service Office
Source: PeerJ Comput Sci. 2025 Feb 17;11:e2672. doi: 10.7717/peerj-cs.2672 (PMC11888853; doi:10.7717/peerj-cs.2672)
Supplement: Supplemental Information 13 [file peerj-cs-11-2672-s013.pdf]

Table A6. Normalized Z-Score scaler result.

DMU	Vin1	Vin2	...	Vin7	Vout1	Vout2	...	Vout6
CQL	-0.75	-0.50	...	-1.09	-0.10	1.04	...	-1.04
EXA	-0.72	0.16	...	-0.14	0.14	-0.44	...	1.41
WOO	-0.73	0.26	...	-0.20	1.99	1.73	...	0.05
...	...	...	...	...	...	...	...	...
WBP	1.28	-0.68	...	-1.00	-0.18	1.72	...	-1.55
