# Supplementary material for: Combination of machine learning and data envelopment analysis to measure the efficiency of the Tax Service Office
Source: PeerJ Comput Sci. 2025 Feb 17;11:e2672. doi: 10.7717/peerj-cs.2672 (PMC11888853; doi:10.7717/peerj-cs.2672)
Supplement: Supplemental Information 14 [file peerj-cs-11-2672-s014.pdf]

Table A7. Minmax scaler result.

DMU	Vin1	Vin2	...	Vin7	Vout1	Vout2	...	Vout6
CQL	0.01	0.18	...	0.08	0.39	0.52	...	0.05
EXA	0.01	0.29	...	0.23	0.41	0.25	...	0.27
WOO	0.01	0.30	...	0.22	0.60	0.64	...	0.14
...	...	...	...	...	...	...	...	...
WBP	0.21	0.15	...	0.09	0.38	0.64	...	0
