# Supplementary material for: Combination of machine learning and data envelopment analysis to measure the efficiency of the Tax Service Office
Source: PeerJ Comput Sci. 2025 Feb 17;11:e2672. doi: 10.7717/peerj-cs.2672 (PMC11888853; doi:10.7717/peerj-cs.2672)
Supplement: Supplemental Information 15 [file peerj-cs-11-2672-s015.pdf]

Table A8. Log transformation scaler result.

DMU	Vin1	Vin2	...	Vin7	Vout1	Vout2	...	Vout6
CQL	-4.93	-1.71	...	-2.53	-0.95	-0.66	...	-3.07
EXA	-4.60	-1.25	...	-1.48	-0.89	-1.39	...	-1.32
WOO	-4.75	-1.19	...	-1.53	-0.51	-0.44	...	-1.94
...	...	...	...	...	...	...	...	...
WBP	-1.54	-1.87	...	-2.37	-0.97	-0.44	...	-inf
