# Supplementary material for: Combination of machine learning and data envelopment analysis to measure the efficiency of the Tax Service Office
Source: PeerJ Comput Sci. 2025 Feb 17;11:e2672. doi: 10.7717/peerj-cs.2672 (PMC11888853; doi:10.7717/peerj-cs.2672)
Supplement: Supplemental Information 16 [file peerj-cs-11-2672-s016.pdf]

Table A9. Cluster and centroid from FCM

Cluster	Centroid
C0	0.106212241679649, 0.379994041367827, 0.48092567989999, 0.353816750556192, 0.227735277731225, 0.573628234029696, 0.277748979143315
C1	0.0996510529546361, 0.214858651444736, 0.277188498642977, 0.224062057264608, 0.132097840088478, 0.361462054305785, 0.184743323223085
C2	0.0089195173116441, 0.101621868876907, 0.0455975123539547, 0.0285237880619545, 0.553745261690683, 0.578091083638992, 0.279831426128825
