# Supplementary material for: Combination of machine learning and data envelopment analysis to measure the efficiency of the Tax Service Office
Source: PeerJ Comput Sci. 2025 Feb 17;11:e2672. doi: 10.7717/peerj-cs.2672 (PMC11888853; doi:10.7717/peerj-cs.2672)
Supplement: Supplemental Information 17 [file peerj-cs-11-2672-s017.pdf]

Table A10. Cluster and members

Cluster	Count	DMU
C0	65	DMU C0, FMO, EDA, FDD, LGN, MGU, LGU, MKU, TXM, CVU, JUB, OST, FGS, BWM, UZL, XDU, TPX, ZEV, EML, WQN, QQR, FDS, JQN, YHS, BBY, FZR, PCU, CCN, WCK, UUB, LOY, YOM, PUW, IGK, AFV, QYI, KSB, TWU, MPN, TNA, PMV, FGT, MKV, OPL, TXJ, UCQ, EPF, ZML, AHA, EWI, ELZ, IAE, NNT, YYK, QNU, BCM, QAO, NZT, RCN, EGZ, PUU, XXV, UWF, XXM, FIA, CWS
C1	149	DMU C1, CQL, WOO, VXK, BEZ, QHN, PER, YFL, EUD, FDQ, STU, YSN, QHD, JPX, MKE, BVA, HYD, ZCZ, NMT, ITE, QNP, CBU, WZZ, BID, FYN, JHB, QYX, PYA, AIY, IOO, YOG, RNY, FLM, MPX, OLF, DKR, EZL, VMY, SWE, UOE, FJN, TFB, BPY, XHH, ICZ, URH, ZND, LUA, MOK, GXQ
C2	138	DMU C2, EXA, FYO, MMR, PGK, PLM, ZXP, KYR, CUP, LFM, YDL, SXY, URG, ZYA, TKM, YZN, OUE, JVA, WUD, PDZ, RJU, MIM, ZDR, AUW, MAK, BZH, KXW, SWM, NCN, YRV, OAQ, GZG, MWI, MNX, KKT, LBI, PLH, ALV, XZM, DCL, GTE, ELQ, ZGO, UBW, AMN, IMW, RYE, SHC, KTC, IJN, AKI, YWG, NLF, GWT, YBU, LHS, FZI, NVW, YHJ, VDP, EOF, KWD, BAS, VVS, YFI, UFE, EOO, VVB, DYV, XYN, VYR, XJB, RLR, WKL, HKS, IJL, TIL, TLS, ORS, NUZ, SBH, OOH, QOQ, DHE, CNW, HKS, GNR, PBB, EMW, EXO, XMB, RDF, MWM, CII, BAV, OQZ, EIG, RUW, HKF, TNX, VJY, GAB, JML, AGE, LIL, HWZ, GIF, OGA, AGQ, ZUH, FCJ, RJE, VWF, NOZ, YHR, BVN, KAS, ZDE, HWM, AGK, TCL, AKY, ACZ, EAR, UML, PMV, SNQ, RFI, BPT, AIJ, JZK, SEL, SPA, XRG, IVN, UPZ, EQN, PJA, CND
