# Supplementary material for: Combination of machine learning and data envelopment analysis to measure the efficiency of the Tax Service Office
Source: PeerJ Comput Sci. 2025 Feb 17;11:e2672. doi: 10.7717/peerj-cs.2672 (PMC11888853; doi:10.7717/peerj-cs.2672)
Supplement: Supplemental Information 18 [file peerj-cs-11-2672-s018.pdf]

Table A11. Input-oriented DEA-BCC result members

CLUSTER	EFFICIENT DMUs	NOT EFFICIENT DMUs
CO	FMO, EDA, MGU, LGU, MKU, TXM, CVU, JUB, OST, FGS, BWM, UZL, XDU, TPX, ZEV, EML, WQN, QQR, FDS, JQN, YHS, BBY, FZR, PCU, CCN, WCK, UUB, LOY, PUW, IGK, TWU, MPN, PMV, MKV, TXJ, UCQ, EPF, AHA, EWI, ELZ, NNT, YYK, NZT, EGZ, XXV, UWF, XXM, CWS	FDD, LGN, YOM, AFV, QYI, KSB, TNA, FGT, OPL, ZML, IAE, QNU, BCM, QAO, RCN, PUU, FIA
C1	CQL, WOO, VXK, BEZ, QHN, EUD, FDQ, STU, YSN, QHD, JPX, MKE, BVA, ITE, QNP, CBU, WZZ, FYN, YOG, RNY, FLM, SWE, UOE, FJN, ICZ, ZND, UXK, KOS, LKM, INI, TRG, CPH, ZXV, NQI, ZWL, XDS, EVL, XSX, BMG, TOA, GJL, SNI, IVW, YIM, HSZ, OZR, OXW, AIQ, DQK, OCM, GIM, QEF, TDT, GPM, YYM, TRB, UZT, FSZ, AIM, DPO, NRA, NEM, TFW, TSU, BPQ, WII, AZU, JJH, EVI, LVZ, BKP, XXW, XAF, BFX, IRI, MRL, MCB, LBV, ANS, CGW, WUK, TJJ, XNH, VAT, OPY, CTF, OXP, QXY, WBP	PER, YFL, HYD, ZCZ, NMT, BID, JHB, QYX, PYA, AIY, IOO, MPX, OLF, DKR, EZL, VMY, TFB, BPY, XHH, URH, LUA, MOK, GXQ, ESI, XAO, LVG, BSU, JKH, KDX, XSX, JTX, AKW, ZAF, CVL, NGY, CPZ, ZEP, GFV, UNF, WXE, KKG, HOB, USH, KJT, MUL, BVY, RPC, GGB, ZOY, ZAD, YJJ, HLU, YCX, REE, RZA, NAX, SGK, LYC, FUU, XWC
C2	EXA, FYO, MMR, PGK, PLM, ZXP, KYR, CUP, LFM, YDL, URG, ZYA, TKM, YZN, WUD, MIM, AUW, BZH, KXW, NCN, YRV, GZG, MWI, MNX, LBI, PLH, XZM, DCL, GTE, ELQ, ZGO, AMN, RYE, YWG, GWT, YBU, LHS, FZI, NVW, VDP, VYR, RLR, WKL, HKS, TIL, TLS, ORS, NUZ, CNW, HKS, GNR, EMW, XMB, RDF, MWM, CII, OQZ, RUW, HKF, VJY, GAFB, AGE, LIL, HWZ, OGA, FCJ, VWF, NOZ, YHR, BVN, KAS, ZDE, AGK, TCL, AKY, ACZ, EAR, PMV, SNQ, RFI, BPT, AIJ, JZK, SEL, SPA, XRG, EQN, PJA, CND	SXY, OUE, JVA, PDZ, RJU, ZDR, MAK, SWM, OAQ, KKT, ALV, UBW, IMW, SHC, KTC, IJN, AKI, NLF, YHJ, EOF, KWD, BAS, VVS, YFI, UFE, EOO, VVB, DYV, XYN, XJB, IJL, SBH, OOH, QOQ, DHE, PBB, EXO, BAV, EIG, TNX, JML, GIF, AGQ, ZUH, RJE, HWM, UML, IVN, UPZ
