# Supplementary material for: Combination of machine learning and data envelopment analysis to measure the efficiency of the Tax Service Office
Source: PeerJ Comput Sci. 2025 Feb 17;11:e2672. doi: 10.7717/peerj-cs.2672 (PMC11888853; doi:10.7717/peerj-cs.2672)
Supplement: Supplemental Information 20 [file peerj-cs-11-2672-s020.pdf]

Table A13. Final Result of the DEA Process

CLUSTER	EFFICIENT DMUs	NOT EFFICIENT DMUs
CO	FMO, EDA, MGU, LGU, MKU, TXM, CVU, JUB, OST, FGS, BWM, UZL, XDU, TPX, ZEV, EML, WQN, QQR, FDS, JQN, YHS, BBY, FZR, PCU, CCN, WCK, UUB, LOY, PUW, IGK, TWU, MPN, PMV, MKV, TXJ, UCQ, EPF, AHA, EWI, ELZ, NNT, YYK, NZT, EGZ, XXV, UWF, XXM, CWS	YOM, OPL, ZML, QNU, PUU, FIA, FDD, LGN, AFV, QYI, KSB, TNA, FGT, IAE, BCM, QAO, RCN
C1	CQL, WOO, VXK, BEZ, QHN, EUD, FDQ, STU, YSN, QHD, JPX, MKE, BVA, ITE, QNP, CBU, WZZ, FYN, YOG, RNY, FLM, SWE, UOE, FJN, ICZ, ZND, UXK, KOS, LKM, INI, TRG, CPH, ZXV, NQI, ZWL, XDS, EVL, XSX, BMG, TOA, GJL, SNI, IVW, YIM, HSZ, OZR, OXW, AIQ, DQK, OCM, GIM, QEF, TDT, GPM, YYM, TRB, UZT, FSZ, AIM, DPO, NRA, NEM, TFW, TSU, BPQ, WII, AZU, JJH, EVI, LVZ, BKP, XXW, XAF, BFX, IRI, MRL, MCB, LBV, ANS, CGW, WUK, TJJ, XNH, VAT, OPY, CTF, OXP, QXY, WBP	PER, YFL, HYD, ZCZ, NMT, BID, JHB, QYX, PYA, AIY, IOO, MPX, OLF, DKR, EZL, VMY, TFB, BPY, XHH, URH, LUA, MOK, GXQ, ESI, XAO, LVG, BSU, JKH, KDX, XSX, JTX, AKW, ZAF, CVL, NGY, CPZ, ZEP, GFV, UNF, WXE, KKG, HOB, USH, KJT, MUL, BVY, RPC, GGB, ZOY, ZAD, YJJ, HLU, YCX, REE, RZA, NAX, SGK, LYC, FUU, XWC
C2	EXA, FYO, MMR, PGK, PLM, ZXP, KYR, CUP, LFM, YDL, URG, ZYA, TKM, YZN, WUD, MIM, AUW, BZH, KXW, NCN, YRV, GZG, MWI, MNX, LBI, PLH, XZM, DCL, GTE, ELQ, AMN, RYE, YWG, GWT, YBU, LHS, FZI, NVW, VDP, VYR, RLR, WKL, HKS, TIL, TLS, ORS, NUZ, CNW, HKS, GNR, EMW, XMB, RDF, MWM, CII, OQZ, RUW, HKF, VJY, GAB, AGE, LIL, HWZ, OGA, FCJ, VWF, NOZ, YHR, BVN, KAS, ZDE, AGK, TCL, AKY, ACZ, EAR, PMV, SNQ, RFI, BPT, AIJ, JZK, SEL, SPA, XRG, EQN, PJA, CND	SXY, OUE, JVA, PDZ, RJU, ZDR, MAK, SWM, OAQ, KKT, ALV, ZGO, UBW, IMW, SHC, KTC, IJN, AKI, NLF, YHJ, EOF, KWD, BAS, VVS, YFI, UFE, EOO, VVB, DYV, XYN, XJB, IJL, SBH, OOH, QOQ, DHE, PBB, EXO, BAV, EIG, TNX, JML, GIF, AGQ, ZUH, RJE, HWM, UML, IVN, UPZ
